# The Stigma of Suicide Survivorship and Related Consequences—A Systematic Review

**DOI:** 10.1371/journal.pone.0162688

**Published:** 2016-09-22

**Authors:** Franz Hanschmidt, Franziska Lehnig, Steffi G. Riedel-Heller, Anette Kersting

**Affiliations:** 1 Department of Psychosomatic Medicine, University of Leipzig, Leipzig, Germany; 2 Institute for Social Medicine, Occupational Health and Public Health, University of Leipzig, Leipzig, Germany; TNO, NETHERLANDS

## Abstract

**Background:**

considerable proportion of the population experiences major life disruptions after losing a loved one to suicide. Social stigma attached to suicide survivors adds to complications occurring in the course of suicide bereavement. Despite its known risks, stigma related to suicide survivors has been sparsely investigated.

**Methods:**

We conducted a systematic literature search in PubMed, Web of Science, PsycInfo and PsyArticles, of studies indexed up through August 2015. Articles were eligible for inclusion if they addressed experiences of stigma in suicide survivors, compared them to other bereavement populations, or investigated stigmatizing attitudes within the public. The search was restricted to English-language studies.

**Results:**

25 records matched inclusion criteria. Study designs were heterogeneous, making comparisons difficult. Results demonstrated that suicide survivors experience stigma in the form of shame, blame, and avoidance. Suicide survivors showed higher levels of stigma than natural death survivors. Stigma was linked to concealment of the death, social withdrawal, reduced psychological and somatic functioning, and grief difficulties. Only one study investigated stigmatizing attitudes towards suicide survivors among the general population.

**Limitations:**

Internal and external validity of the studies was restricted by a lack of valid measures and selection bias.

**Conclusions:**

More methodologically sound research is needed to understand the impact of stigma on suicide survivors’ grief trajectories and to separate it from other grief aspects. Clinicians and grief-counselors as well as the public should be educated about the persistent stigma experienced by suicide survivors.

## 1. Introduction

A suicide survivor is defined here as “someone who has lost a significant other to suicide” [1]. Recent estimates indicate that the number of individuals affected by a suicide is considerable. For every suicide, there are approximately 18 individuals experiencing major life disruptions in the course of suicide survivorship [2]. Given an annual rate of 800,000 suicides worldwide [3], up to 14.4 million people might be intimately affected by suicide bereavement every year.

Although suicide bereavement appears to largely resemble grieving after other types of loss in terms of symptom severity [4], there is indication that some suicide survivors are at an increased risk of developing depression, suicide ideation, and a pathological grief reaction, defined as *prolonged grief disorder* or *complicated grief* in the bereavement literature [5–7]. Complications occurring in the course of grieving appear to be related to a range of qualitative themes common in suicide bereavement. Thus, suicide survivors face the burden of finding reasons to explain the death and suffer from feelings of shame about the cause of the death, guilt for not being able to prevent the death, blame directed towards self and others and abandonment/rejection by the deceased [7–10]. Another factor that characterizes suicide bereavement and that may greatly complicate the grieving process after suicide is the social stigma attached to suicide survivors [7,10–13].

Stigmatization of suicide survivors can be traced back to early historic periods when family members of suicide were faced with being denied a proper burial of the deceased, property confiscation, and excommunication from the community [1,12]. Although such cultural practices have ceased to exist, there is evidence that negative attitudes towards those bereaved by suicide prevail and that stigmatization has taken more subtle forms of isolation and shunning [8,14–17]. Additionally, the ongoing stigmatization of suicide and associated mental illnesses provides further indication that stigma is a persistent part of the grief experiences of suicide survivors [13,18–21].

In recent years, scientists have come to understand stigma as a social process that involves labeling, stereotyping, and rejecting human differences in order to exert social control [22,23]. Theoretical conceptualizations further distinguish between two interrelated dimensions across which stigma can be assessed: public stigma and self-stigma [24]. Public stigma refers to “the phenomenon of large social groups endorsing negative stereotypes about and acting against a stigmatized group” [25]. Self-stigma describes a process whereby stigmatized individuals perceive social devaluation (perceived stigma), experience actual enactments of stigma in the form of discrimination (enacted stigma), or internalize negative attitudes of others, resulting in an adverse self-image characterized by shame, guilt, maladaptive behaviors, and/or stereotype endorsement (internalized stigma) [24,26].

This conceptualization illustrates how stigma might interfere with the grieving process of suicide survivors. Negative attitudes within the public can be conveyed to the survivors through various pathways, such as attribution of blame by the survivor’s social environment, gossip or negative media portrayal of the deceased [14,27,28]. If suicide survivors internalize these negative attitudes, this could exacerbate existing feelings of shame, self-blame and/or guilt. In order to avoid stigmatization, suicide survivors might also engage in maladaptive behaviors, such as concealment of the cause of death [7]. Research among members of stigmatized groups has shown a multitude of other adverse outcomes of stigma. It has been linked with psychological distress, reduced self-esteem, poor quality of life, a reduction of social support networks, and delays in seeking care from healthcare professionals [29–32].

Although the literature has identified stigma as an important factor in suicide bereavement, so far, it has been only sparsely investigated. While few existing reviews have focused on stigma associated with suicide survivors [12,13], non-systematic modes of data collection limit conclusions and several new studies have been published since the publication date of the most recent review in 2008. Especially the psychological consequences of stigma for affected suicide survivors remain understudied, despite findings from related research fields suggesting serious negative effects of stigma. Thus, this review aims at providing a critical overview on the current state of research on suicide survivor stigma and to investigate the influence of stigma on grief trajectories and survivors’ well-being.

## 2. Methods

This review was conducted based on PRISMA guidelines [33]. We did a systematic literature search in PubMed, Web of Science, PsycInfo, and PsyArticles of studies indexed up through August 2015. Search terms included variations of the keywords “stigma” and “suicide survivor” using the search strategies described in Table 1.

**Table 1:** Database search strategies.

Database	Search strategy	Results
Web of Science (all databases)	Search field: Topic; Search term: (stigma* OR prejudice OR judgment* OR discrimination OR stereotyp* OR attitude* OR opinion OR perception*) AND suicid* AND (survivor* OR bereav* OR grief); Limits: Restricted to articles, abstracts, reviews or case reports.	403
PubMed	Search field: All fields; Search term: (stigma* OR prejudice OR judgment* OR discrimination OR stereotyp* OR attitude* OR opinion OR perception*) AND suicid* AND (survivor* OR bereav* OR grief); No limits used.	324
PsycInfo	Search field: All fields; Search term: (stigma* OR prejudice OR judgment* OR discrimination OR stereotyp* OR attitude* OR opinion OR perception*) AND suicid* AND (survivor* OR bereav* OR grief); Limits: Books and dissertations excluded.	276
PsycArticles	Search field: All fields; Search term: (stigma* OR prejudice OR judgment* OR discrimination OR stereotyp* OR attitude* OR opinion OR perception*) AND suicid* AND (survivor* OR bereav* OR grief); No limits used.	24

The database search was restricted to empirical human research articles written in English and published in peer-reviewed journals. After removing duplicates, two independent researchers (FH and FL) identified potentially relevant articles by screening titles and abstracts and then retrieving articles in full-text for a detailed evaluation against inclusion criteria. They also checked reference lists of relevant articles to identify articles not covered by the database search. Both quantitative and qualitative studies were included. Articles were eligible for inclusion if they addressed self-stigma or public stigma related to suicide survivors either by explicitly measuring stigma quantitatively, or by reporting stigma as a major qualitative research finding. If studies involved controls of other bereavement populations, each of those control groups had to be homogenous regarding the type of loss experienced (e.g. natural loss, accidental loss). Studies were excluded if: (1) they investigated the public perception of suicide survivors without explicitly using a measure of stigmatization; (2) focused on bereavement after an assisted suicide or (3) were intervention studies, case studies, reviews, commentaries, theory papers or published as books or book chapters. Data was extracted from eligible studies according to the following scheme: Sample, Design, Participants, Stigma Conceptualization, Main Outcome Measure(s), Main Findings, and Bias.

Due to the high diversity of research designs and methodology applied in the studies included, a narrative synthesis of data was deemed appropriate. Following recent guidelines [34] we did a qualitative assessment of the methodological quality of studies within the text rather than using summary scores of quantitative checklists.

## 3. Results

### 3.1 Study characteristics

**Fig 1:**
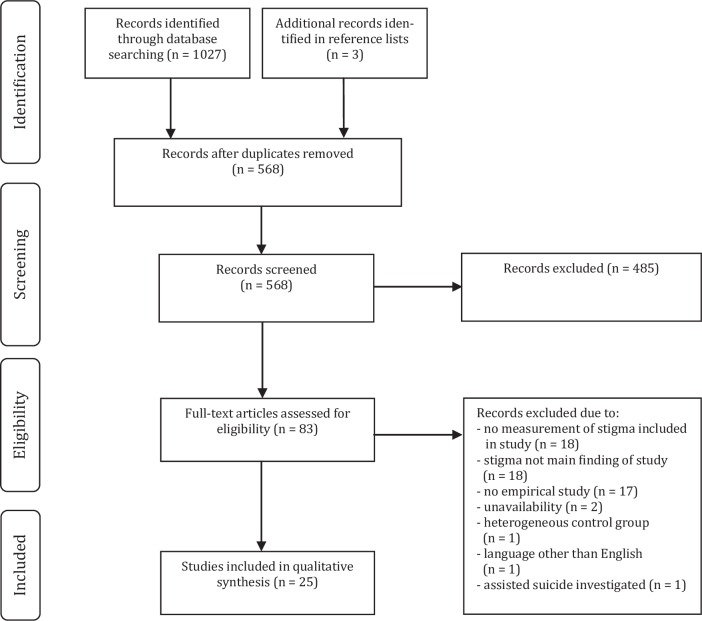
PRISMA Flow Diagram.

Fig 1 illustrates the course of the literature search. After removing duplicates, the database search yielded a total of 568 articles. Of these, 22 articles met inclusion criteria. Three of these articles were based on the same study but, for our purposes, were counted as separate studies because the composition of the samples varied [15,35,36]. Three additional studies were identified by searching the reference lists of included articles, resulting in a total of 25 included studies.

Table 2 and Table 3 provide an overview of the studies’ characteristics. The majority of the studies had been published in 2002 or later (N = 15), while the earliest study dated from 1983. Fourteen studies were quantitative and eleven were qualitative. Most studies provided descriptive data on self-stigma among suicide survivors (N = 15). Other studies investigated associations between stigma and grief, psychosomatic functioning and sociodemographic variables (N = 7). Eight studies compared the stigma experiences of suicide survivors to those of other bereavement populations using controlled study designs. Only one study was found that examined public stigma towards suicide survivors.

**Table 2:** Summary of quantitative studies on levels and correlates of self- and public stigma.

Study	Study design and location	Relationship to deceased	Main outcome measures	Results
Wojtkowiak et al. [37]	Cross-sectional; 142 SDS (Belgium, Germany, Netherlands)	Partner, parent, sibling, children, someone other	Self-stigma (GEQ), grief-related and somatic functioning	• Stigma associated with suicide-specific grief reactions (e.g. guilt, *r* = .41), somatic reactions (*r* = .35), overall grief difficulties(*r* = .76).
Feigelman et al. [35]	Controlled; 462 SDS, 24 SND, 37 SAD, 48 SCDO (USA)	Parent	Self-stigma (SGS)	• SDS = SCDO
Feigelman et al. [15]	Controlled; 462 SDS, 24 SND, 54 STD (USA)	Parent	Self-stigma (SGS), grief difficulties, depression, suicide ideation	• SDS = SND, SDS = STD • Stigma predicted grief difficulties (*beta* = .42), depression (*beta* = .18) and suicide ideation (*beta* = .09).
Feigelman et al. [36]	Cross-sectional; 401 SDS (USA)	Parent	Self-stigma (SGS), grief difficulties	• Stigma predicted grief difficulties (*beta* = .210).
McMenamy et al. [38]	Cross-sectional; 63 SDS (USA)	Parent, children, sibling, spouse, grandparent, friend	Self-stigma (SGS)	• 42% moderate to high levels of shame; 40% social isolation and withdrawal of friends; 16% experienced gossip/blame.
Houck [39]	Controlled; 50 SDS, 50 SAND, 50 STD (USA)	Children, spouse, sibling, parent, lose friend, life-partner, extended family member	Self-stigma (GEQ)	• STD > SDS, SND.
Harwood et al. [40]	Controlled; 45 SDS, 46 SND (UK)	Children, friend, spouse, sibling, other relative	Self-stigma (GEQ)	• SDS > SND.
Silverman et al. [41]	Controlled; 9 SDS, 12 SAND, 9 SUND, 16 SAD, 9 SH (USA)	“loved one”	Self-stigma (GEQ)	• SDS > SUND, SAND • SDS > SH, SAD.
McIntosh & Kelly [28]	Controlled; 40 SDS, 63 SND, 71 SAD (USA)	Parent, sibling, spouse, offspring, other relative, close friend	Self-stigma (SGS)	• SDS, SAD > SND • 87% felt stigmatized to some degree, 53% experienced gossip, 35% concealed death, 13% felt avoided, 3% were blamed.
Barrett & Scott [42]	Controlled; 14 SDS, 15 SAD, 15 SUND, 13 SAND (USA)	Spouse	Self-stigma (GEQ)	• SDS > SAND • SDS = SUND, SAD.
Cleiren et al. [43]	Controlled; 91 SDS, 93 SAD, 125 SND (Netherland)	Spouse, parent, sibling, adult children	Self-stigma (SGS)	• T1: SDS = SND, SAD • T2: SDS = SND, SAD.
McIntosh & Wrobleski [44]	Cross-sectional; 141 SDS (USA)	Children, parent, sibling, spouse	Self-stigma (SGS), psychological and somatic functioning	• 85% felt guilty, 36% felt avoided; 20% were blamed; 15% concealed death; 4% experienced gossip. • Stigma associated with suicide specific aspects of grief (e.g. guilt *r* = .29). • Stigma associated with number of personal symptoms (e.g. headaches, stomach pain, panic attacks, and concentration difficulties) (*r* = .40).
Solomon [45]	Cross-sectional; 90 SDS (USA)	Sibling, spouse, parent, children, friend, second-degree relative	Self-stigma (SGS)	• 31% felt stigmatized.
Scocco et al. [1]	Controlled; 282 people from general population, 113 psychiatry patients, 75 SDS (Italy)	Not reported	Public stigma (STOSASS)	• Moderate levels of stigmatizing attitudes towards suicide survivors within the general population.

SDS, suicide survivors; SAD, survivors of accidental death; SCDO, survivors of child death by drug overdose; SND, survivors of natural death; STD, survivors of traumatic death; SAND, survivors of anticipated natural death; SUND, survivors of unexpected natural death; SH, survivors of homicide. GEQ, *Grief Experience Questionnaire*; SGS, self-generated scale; STOSASS, *Stigma of Suicide and Suicide Survivor scale*.

**Table 3:** Summary of qualitative studies on subjective experiences of stigma among suicide survivors.

Study	Study design and location	Relationship to deceased	Topic of exploration	Results
Chapple et al. [46]	Interviews; 40 SDS, 40 STD (UK)	Parent, children, sibling, partner, other relatives or friend	Exploring the circumstances that impede bereavement after sudden traumatic death	• SDS felt blamed and ostracized by others • Some SDS highlighted the necessity to resist stigma.
Gall et al. [47]	Interviews; 11 SDS (Canada)	Children, parent, second-order relative, close friend	Understanding the experiences of suicide bereaved individuals	• SDS sensed others discomfort and were unsure of reactions they might receive. • Direct discrimination only in one case. • Most SDS rejected stigmatization in honor of deceased.
Tzeng et al. [48]	Interviews; 15 SDS (Taiwan)	Children, sibling, grandchildren, second-order relative	Adjustment to suicide survivor stigma in Chinese culture	• SDS kept low profile funeral due to stigma. • Stigma internalized as a deep sense of shame in SDS. • SDS concealed cause of death and suppressed emotions.
Thrift & Coyle [49]	Interviews; 7 SDS (UK)	Mother	Exploring the impact of child suicide on maternal identity	• Participants felt stigma as negative distinctiveness from others, posed a potential threat to maternal identity. • Stigma manifested as being/feeling avoided.
Avrami [50]	Interviews; 30 SDS^1^ (Israel)	Children	Exploring the impact of parental suicide on surviving children in Israeli culture	• Participants felt stigma from association with mental illness, linked to concealment of death and memories.
Fielden [51]	Interviews; 6 SDS (New Zealand)	Parent, sibling	Grief experiences of close family members bereaved by suicide	• Stigma associated with social withdrawal. • Avoidance of survivors linked to stigmatization of suicide, but also to disability of others to provide support.
Biddle [27]	Interviews; 16 SDS (UK)	Parent, spouse, sibling, children	Documenting suicide survivors’ experiences of suicide inquests in the UK	• Shame and stigma major theme in SDS’s experience of coroner’s inquest. • SDS felt publicly judged and condemned as criminals and social outcasts.
Clark & Goldney [52]	Support group discussions; 97 SDS (Australia)	Parent, partner, sibling, children, second-order relative, friend	Grief reactions and recovery in a support group for people bereaved by suicide	• Participants felt stigma from social networks, media and institutions. • Stigma manifested as rejection, blame and gossip, linked to long-term deception.
Van Dongen [53]	Interviews; 35 SDS (USA)	Parent, adult children, sibling, spouse	Social context of post-suicide bereavement	• SDS felt rejected by others, was perceived as stigmatizing. • Rejection evident through absence of behavior rather through direct behavior.
Demi & Howell [54]	Interviews; 17 SDS (USA)	Children, sibling	Exploring long-term effects of the suicide of a parent or sibling	• Stigma expressed as feeling ashamed or tainted. • Stigma resulted in wish to conceal/deny the death.
Dunn & Morrish-Vidners [55]	Interviews; 24 SDS (USA)	Spouse, parent, sibling, children	Exploring psychological and social dimensions of suicide bereavement	• Participants reported persistent feelings of stigmatization. • Suicide associated with mental illness stigma. • Stigma linked to concealment of emotions and social isolation

SDS, suicide survivors; STD, survivors of traumatic death.

^1^Personal communication with the author

Sample sizes varied from 55 to 571 participants in the quantitative studies, and from 6 to 97 participants in the qualitative studies. Samples were recruited in 10 different countries, with the majority of samples located in the U.S. (N = 13), the U.K. (N = 4), or other Western countries (N = 6). One study was conducted in Israel and one in Taiwan. In 12 of the 25 studies, participants were spouses or first-degree relatives of the deceased (children, siblings, parents). Other studies included survivors with mixed relationships to the deceased (e.g. parents, friends, second-degree relatives). The vast majority of the studies involved non-representative convenience samples.

### 3.2 Assessment of suicide survivor stigma

Measurements of stigma related to suicide survivors lacked conceptual clarity, resulting in the use of a wide range of assessment tools. In 8 out of 14 quantitative studies, levels of self-stigma in suicide survivors were assessed with non-validated self-generated items or scales. They included items regarding survivors’ exposure to and perception of negative responses from their social environment such as avoidance, relationship strain, insensitivity or blame by others, and negative feelings about self like shame or guilt. These measures showed considerable variation in their operationalization of stigma, making comparisons across studies difficult. However, five studies applied the standardized Grief Experience Questionnaire (GEQ) [56] that includes a *Stigmatization* subscale. The GEQ was specifically developed to assess various components of suicide bereavement in affected individuals, with the stigmatization subscale reflecting “the common suggestion that suicide negatively and permanently marks the suicide survivor as different from other survivors” [56]. While internal consistency (.68 < alpha < .89) of subscales and discriminant validity was shown, the factorial structure of the original version could only partly be replicated in further analysis [57].

One study developed a standardized instrument that can be used to assess stigma among the general population as well as suicide survivors. The STOSASS (Stigma of Suicide and Suicide Survivor) captures respondents’ perceptions of how “most people” would think of and act towards a suicide survivor, for example with regard to the survivor’s intelligence, trustworthiness, and eligibility as a close friend or employee [1]. The scale proved to have good reliability both in terms of internal consistency (0.80 < lambda2 < 0.89) and test-retest stability (ICC = 0.85) while factorial analysis produced a meaningful solution.

### 3.3 Self-Stigma among suicide survivors

Prevalence rates and subjective experiences of stigma among suicide survivors were investigated in 4 quantitative [28,38,44,45] and 11 qualitative studies (Table 3). Although these studies did not directly address the multi-dimensionality of self-stigma, we categorized results according to the definitions of perceived stigma, internalized stigma, and enacted stigma outlined above in the introduction section. These studies also reported on survivors’ strategies for managing stigma.

#### 3.3.1 Perceived stigma

In one quantitative study that used a dichotomous measure of stigma, 31% of respondents reported that they felt a general stigma surrounding their status as suicide survivors [45]. Another quantitative study found that as much as 87% of suicide survivors felt stigmatized to some degree, with 23% reporting strong feelings of stigmatization [28]. Differences in study findings might be related to mode of measurement, as the latter study used a continuous measure of stigma.

In the qualitative studies, participants also frequently described feeling stigmatized, which was experienced as a negative distinctiveness from others [46,47,49,55]. Qualitative results further provided indication on sources of stigmatization. Thus, stigma was perceived from the ongoing association between suicide and mental illness, which survivors felt discredited not only the deceased but also themselves [50,52,55]. Based on accounts of participants from Australia, the formerly illegal status of suicide as well as the church’s negative attitudes towards suicide and popularly held spiritual beliefs induced feelings of stigmatization in suicide survivors [52]. Tzeng et al. [48] found that suicide is not viewed as an individual, but a collective failure to attend to group norms in Chinese culture. Consequently, suicide brings dishonor and stigma not only to the deceased, but also to the associated family as a whole.

#### 3.3.2 Internalized stigma

In two quantitative studies, internalized stigma was operationalized as feelings of guilt and shame, which were reported by 85% and 42% of participants, respectively [38,44].

Results from qualitative studies also showed that perceived stigmatization went along with a deep sense of shame and embarrassment in suicide survivors [27,48,50,52,54,55]. Participants described feeling tainted, degraded or awkward in the aftermath of the suicide, suggesting that they had adopted devaluing social attitudes towards their status as suicide survivors [54,55]. However, there was some evidence that survivors had developed negative feelings towards themselves independent of other people’s attitudes [55].

#### 3.3.3 Enacted stigma

Study results suggest that actual enactments of stigma towards suicide survivors were mainly subtle and emerged in the form of avoidance and rejection. The majority of quantitative studies found that about one third of suicide survivors (36% and 40%) felt avoided by people in their social network [38,44], while in one study 13% of participants reported feeling avoided [28]. The number of suicide survivors reporting strong negative reactions of others was generally lower but varied considerable, with 3% to 53% having had experienced stigma in the form of gossip or blame [28,38,44].

Results of qualitative studies indicated that stigma contributes to a lack of social norms on how to deal with suicide and suicide survivors. Participants reported sensing other people’s fear and discomfort in social interactions and had experienced ostracism, avoidance and rejection [46,47,49,51,53,55]. Parallels between stigma and attributions of responsibility were evident in the qualitative studies. Participants described being blamed for the suicide by family members, friends, and professionals, which was experienced as stigmatizing [46,51,52]. However, it appeared that in some cases survivors’ perception of blame was unrealistic and exaggerated [52]. Also within the qualitative studies, few participants reported having experienced strong negative reactions of others, which included being subject to gossip, or being met with anger and resentment by other people when talking about the suicide [52,55]. One UK study found that suicide survivors experience stigmatizing treatment during legal trials following a suicide, for example in the form of privacy invasion or negative media portrayal [27].

#### 3.3.4 Stigma management

The reviewed studies indicated that suicide survivors react with concealment and social withdrawal to perceived and internalized stigma. In the quantitative studies, 15% to 35% of participants indicated that they had concealed their loss experience or did not want to talk about the death [28,44].

Concerning qualitative results, there was some evidence that stigma led survivors to conceal the cause of the death, withdrew from social interactions or suppress their grief-related emotions [48,50,51,52,54,55]. This in turn appeared to deprive survivors of the support they needed [48,55]. In two studies, participants held low profile funerals and/or attempted to hide the memory of the deceased in order to avoid the profound stigma associated with suicide in Israeli and Chinese culture [48,50]. However, some studies suggested that the social withdrawal of survivors was in part self-imposed as survivors needed time to cope with their grief [53,55]. There was also, albeit limited, qualitative evidence that some survivors rejected stigmatization, for example by actively confronting others with their grief [46,47].

### 3.4 Stigma correlates

Seven studies examined the associations of stigma with grief difficulties and sociodemographic variables [1,15,35–37,43,44]. In detail, studies provided information on associations between self-stigma and overall grief difficulties, suicide specific aspects of grief, impairments in psychosomatic functioning and sociodemographic variables.

Wojtkowiak et al. [37] used a modified version of the GEQ to investigate the grief experiences of adults bereaved by suicide. Inter-subscale correlations showed that a higher sense of stigma in survivors was significantly correlated with greater overall grief difficulties, suicide specific aspects of grief (such as shame, guilt or search for explanation), a tendency toward self-destructive behaviors, and increased somatic reactions. However, validity of results was limited by the absence of separate measures of grief variables. Using a self-generated stigma scale, Feigelman and colleagues [15,36] found that a high sense of stigma significantly predicted more overall grief difficulties among parents bereaved by suicide. Higher levels of stigma were also significantly associated with increased depression and suicidal ideation. The authors additionally investigated the influence of stigma on levels of pathological grief as measured by the Inventory of Complicated Grief [58] and found that stigma significantly contributed to explaining variance in pathological grief among participants. Analyses were controlled for loss-related and individual characteristics. McIntosh and Wrobleski [44] explored the grief experiences of individuals bereaved by the suicide of a close family member with a range of self-generated measures. Results showed that the number of stigmatizing events experienced was significantly correlated with greater amounts of guilt, anger and problematic thoughts about the death as well as the presence of symptoms including somatic (e.g. headaches, stomach pain) and psychological issues (e.g. panic attacks, concentration difficulties).

Gender, age and relationship to the deceased were found to influence participant’s experiences of stigma. Female participants were at a higher risk of experiencing stigma [1,35,37]. Results further indicated that younger suicide survivors experience more stigma [44]. Children, siblings and spouses of the deceased appeared to be specifically at risk of experiencing stigma, with parents feeling less stigmatized [43,44].

### 3.5 Stigma compared across different survivor groups

A total of 8 quantitative studies compared self-stigma in suicide survivors to a variety of other survivor groups. Controls included survivors of loss by natural death (unexpected and anticipated), accidental death, homicide, child drug overdose, and HIV/AIDS (Table 2). In order to provide a meaningful overview on the literature, results of controlled studies in this review were grouped into two categories: the first category is comprised of suicide survivors and survivors of loss by natural death; and the second category is comprised of suicide survivors and survivors of deaths linked to other traumatic or stigmatized conditions (i.e. accident, homicide, child drug overdose, and HIV/AIDS). Differences and non-differences for each comparison made, time point, and measure of stigma were treated as separate outcomes. If applicable, only total scores of stigmatization scales were considered. Studies often included multiple comparisons and assessments, and thus contributed multiple times to both of the aforementioned categories.

#### 3.5.1 Suicide survivors vs. survivors of loss by natural death

Self-stigma in suicide survivors as compared to survivors of loss by natural death was measured 10 times across 6 studies [15,28,40–43]. In 6 assessments, suicide survivors reported a significantly greater sense of stigma, whereas in 4 assessments, no difference was found.

Harwood et al. [40] studied grief experiences and needs in survivors of the death of an older person. Analysis of the GEQ revealed that suicide-bereaved participants experienced more stigma than participants bereaved by natural death. However, the distribution of degrees of kinship to the deceased differed between suicide survivors and controls, potentially biasing results. Silverman et al. [41] compared bereavement following suicide loss with bereavement following anticipated and unexpected natural deaths among university students. The authors found that participants bereaved by suicide experienced more stigmatization as measured by the GEQ than both groups of natural death survivors. However, group sizes were small, and analyses were not controlled for the observation that suicide-bereaved participants felt closer to the deceased than survivors of natural death. In another study with bereaved university students, McIntosh and Kelly [28] assessed self-stigma with a self-generated scale. Results revealed that suicide survivors reported feeling more stigmatized and experienced more stigmatizing events than natural death survivors. This study’s data might have been influenced by recall bias, as survivors were asked to remember stigmatizing events over an average time period of 5.93 years (SD = 6.8). Studying grief reactions among bereaved spouses, Barrett and Scott [42] found that experiences of stigma were related to how expected the natural death had been. Analysis of the GEQ-scores showed that suicide survivors experienced more stigma compared to survivors of anticipated natural loss, but not more than survivors of unexpected natural loss.

Feigelman et al. [15] investigated self-stigma among parents bereaved by child suicide. Using a self-generated scale, they found no differences in survivors’ experiences of stigma between parents who lost a child to suicide and parents who lost a child to natural death. In a longitudinal study, Cleiren et al. [43] examined the bereavement trajectories of bereaved first-degree family members. They compared exposure to loss from suicide to loss after a long-term illness, at 4 and 14 months after the death. Using a self-generated scale to assess survivors’ experiences of stigmatizing social responses, they found no differences between bereavement groups at any time point.

#### 3.5.2 Suicide survivors vs. survivors of traumatic loss

Self-stigma in suicide survivors, as compared to survivors of other traumatic or stigmatized loss, was measured 8 times across 6 studies [15,35,39,41–43]. Six assessments observed either no difference or a greater sense of stigma in the comparison group, whereas two assessments found that suicide survivors experienced significantly more stigma.

In two publications, Feigelman and colleagues [15,35] used a self-generated measure to compare self-stigma in a sample of parent survivors of child suicide to parent survivors of child death by traumatic circumstances (e.g. accident, homicide) and drug overdose. Results showed that suicide survivors did not experience more stigma than other survivor groups. Houck [39] contrasted grief reactions in individuals bereaved by HIV/AIDS, suicide, and cancer. Scores on the stigmatization subscale of the GEQ indicated that participants bereaved by HIV/AIDS experienced more stigma than both survivors of loss by suicide and cancer. In the longitudinal study by Cleiren et al. [43], no differences regarding experiences of stigmatizing social responses were observed between suicide survivors and survivors of loss by traffic fatalities at either 4 or 14 months following the loss. Likewise, Barrett and Scott [42] found no greater sense of stigma in suicide survivors than in survivors of loss by accident.

On the other hand, Silverman et al. [41] found that suicide survivors reported greater stigma than both survivors of loss by accident and loss by homicide.

### 3.6 Public stigma towards suicide survivors

Our literature search identified only one study investigating stigmatizing responses of the public to suicide survivors. Scocco et al. [1] used a standardized scale to compare negative attitudes towards suicide-bereaved individuals in the general population to those held by psychiatric patients and suicide survivors. Analysis of responses revealed that stigma scores did not differ between the groups, with participants generally expressing moderately negative attitudes towards suicide survivors.

## 4. Discussion

Although the literature has identified stigma as an important factor in suicide bereavement, the results of this systematic review revealed that there is a paucity of research on manifestation and consequences of suicide survivor stigma. The available studies showed considerable variation with regard to study design and stigma measures, making comparison across studies difficult. However, results demonstrate that stigma can be a serious concern for a significant proportion of suicide survivors, as opposed to survivors of a natural loss. Suicide survivors experienced stigma within their social networks as well as from legal and religious institutions in different cultural settings. Stigma went along with social isolation and was internalized as intense feeling of shame by suicide survivors. However, little is known about factors that influence survivors’ experiences of stigma. Previous research has highlighted the role of relationship to the deceased in reactions to a loss, with parents who lost a child usually encountering the greatest difficulties [43]. Contrary to these findings, the included studies found that parent suicide survivors experience less stigmatization than other immediate family members. It is possible that these discrepant finding were produced by differences in quality of the kinship relation, which studies did often not take into account. Further, distinct information regarding feelings of stigma in more distant associates of the deceased such as co-workers were lacking in the included studies. Some suicide survivors had developed strategies to resist stigmatization, but the psychological mechanisms of these strategies remain unknown. Hence, more research is needed to clarify how experiences of stigma vary as a function of individual and contextual factors, in order to identify specific risk populations. A deeper insight into stigma management strategies might help other survivors building up individual resistance and coping with negative feelings in the aftermath of a suicide.

The reviewed studies further revealed that suicide survivors’ experiences of stigma are associated with grief difficulties as well somatic and psychological impairments such as depression and suicide ideation. This evidence tentatively supports the assumption that stigma complicates the grieving process of suicide survivors [8,12]. Moreover, stigma may contribute to heightened suicide risk in survivors of suicidal deaths [5,7]. None of the quantitative studies investigated the underlying mechanisms that mediate the relationship between stigma and complications occurring in the course of grieving. However, the results indicate that suicide survivors frequently conceal the death from others, withdraw from social interactions, and suppress their emotions to manage stigma. Studies involving members of other stigmatized groups have demonstrated that concealment is associated with intrusive thoughts, increased psychological distress, anxiety, and depression [59–61]. There was also some indication that concealment and secrecy deprived suicide survivors of support from others, potentially exacerbating existing grief difficulties. Other studies have shown that suicide survivors who experience a lack of social support are more likely to experience unresolved grief, depression and separation anxiety [62,63]. Given the consistent link between stigma and avoidance behaviors, stigma might also pose a barrier for burdened suicide survivors to seek professional help. Indeed, Wilson and Marshall [64] have shown that there is a significant gap between the need for support in people bereaved by suicide and the provision of professional help.

However, the influence of stigma on grief trajectories and suicide survivors’ support networks needs further methodologically sound research as many of the studies included in this review were flawed due to widespread use of non-validated measures, cross-sectional designs and unadjusted correlational analysis. Especially investigations on the relation between stigma and complicated grief/prolonged grief disorder might advance understanding of the processes that make suicide survivors vulnerable to the development of a pathological grief reaction [6].

This review further provides a differentiated view on the assumption that stigma is a specific characteristic of suicide bereavement, as suggested by some research [4]. While results indicated a tendency for suicide survivors to experience more stigma than natural death survivors, differences disappeared when compared to other traumatic death survivors. These results are converging with a line of research proposing that suicide bereavement and bereavement by other types of traumatic losses produce similar reactions [7,8]. The exact reasons for similarities in experiences of stigma among suicide survivors and survivors of other traumatic deaths remain unclear, but several explanations might apply. It is possible that suicide survivors and other traumatic death survivors evoke fear, avoidance responses, and false attribution of responsibility because they confront others with their own inability to control fundamental issues of life and death [15,55]. In addition, the absence of clear social norms on how to interact with survivors of suicide and traumatic deaths might contribute to similar patterns of insensitivity on the part of the people in survivors’ social networks, which can be perceived as stigmatizing by survivors [15,46].

Understanding the interaction between survivors’ individual emotional reactions to the suicide and surrounding social processes poses another important challenge for future research. Study results suggest that survivors’ emotional reactions to the suicide influence their sense of stigma and strategies for coping with the loss. Suicide survivors have been shown to frequently suffer from negative feelings like shame, guilt, anger and blame [7,8]. While perceived stigma appears to feed into these feelings, it is also possible that suicide survivors project their negative emotions on their social environment, leading to a biased perception of others’ behaviors and attitudes. Some studies further indicate that the social isolation of survivors is not only a reaction to stigma but in part self-imposed to give room for grieving. However, these complex issues cannot be definitely resolved on the basis of the current evidence. The reviewed studies often lacked a clear definition and conceptualization of stigma, which went along with the use of unspecific stigma measures. Such an approach makes it difficult to assess the relationship between more stigma-specific grief components, such as perceived negative judgment, and related emotional and social processes, such as shame, guilt or concealment of the death. The observed lack of stigma conceptualizations also hinders the development of theoretical frameworks that are necessary to systematically advance knowledge on the role of stigma in the context of suicide bereavement.

Assessing the adequacy of suicide survivors’ perception of stigma was further hampered by the absence of studies on stigmatizing attitudes towards suicide survivors within the public. Only one study met inclusion criteria with results indicating that stigmatizing attitudes are indeed common among the general population. This result supports the findings of a larger body of research examining general public perception of and biases towards suicide survivors (see [8], [14], [17] for reviews). These studies suggest that suicide survivors are perceived more negatively than survivors of other modes of death, such as that they are viewed as more psychologically disturbed, are blamed more for the death and are less likeable (e.g. [65–67]). Thus, there is indication that suicide survivors’ perception of stigma might actually be based on adequate evaluation of other’s attitudes, although the limited evidence from this review allows only cautious inference. While more research on negative attitudes and stereotypes among close social circles of suicide survivors, as well as their community and the general population is needed, it is also important to understand how these attitudes translate into direct behavior towards suicide survivors. Observing interactions of suicide survivors within their social network might help to understand how stigma as well as individual emotional reactions to a loss by suicide contribute to the cycle of strained social interactions and isolation experienced by suicide survivors [14,68,69].

### 4.1 Limitations

Studies included in the review had a range of methodological shortcomings. The vast majority of studies included samples that were conveniently recruited, e.g. from support groups, which limits generalizability of findings. The internal validity of the quantitative studies was further restricted by the widespread use of unstandardized, non-validated assessment instruments.

This review also has some limitations. To ensure reporting quality, only studies published in peer-reviewed journals were included. This may have led to the exclusion of relevant gray literature. In order to provide a comprehensive overview of the literature, there were no publication date restrictions placed on the studies included. Thus, it is possible that some of the earlier studies may no longer adequately reflect current experiences of stigma among suicide survivors. Furthermore, the search was restricted to articles published in English.

### 4.2 Conclusion

While the degree of stigmatizing attitudes towards suicide survivors within the public needs to be more firmly established, studies reliably indicate that feelings of stigma are a relevant part of the subjective bereavement experience of suicide survivors. Stigma has the potential to shame and silence suicide survivors; it can isolate survivors within their social network and is associated with grief difficulties and psychological impairments. However, the impact of stigma on the development of grief complications needs further investigation using validated measurement instruments, longitudinal research designs, and representative samples. Given the complex interactions between social processes and individual emotional responses to a loss by suicide, it is important to separate stigma from other grief reactions. Applying measurements that clearly differentiate between the different dimensions of self-stigma (perceived, internalized, and enacted) and public stigma could be an important first step. More research is also necessary to identify the needs of suicide survivors in the face of stigma, and in turn design effective interventions. There is some evidence that low-threshold services such as peer-counseling or online support groups are suitable for addressing the needs of stigmatized survivors [36,70]. Providing education about the underlying psychological mechanisms of suicide in schools, in the workplace and among peer-groups has been shown to positively affect attitudes towards suicidal people and might be adapted to normalize suicide bereavement in the eyes of the public [71,72]. Employees in healthcare settings who work with suicide survivors should reflect on their own attitudes towards suicide and survivors, sensitively discuss issues of blame and guilt, and provide a non-judgmental space in which survivors can give individual meaning to their loss-experience.

## Supporting Information

S1 PRISMA Checklist (DOC)
